# Therapeutic potential of an anti-angiogenic multimodal biomimetic peptide in hepatocellular carcinoma

**DOI:** 10.18632/oncotarget.21148

**Published:** 2017-09-21

**Authors:** Mustafa A. Barbhuiya, Adam C. Mirando, Brian W. Simons, Ghali Lemtiri-Chlieh, Jordan J. Green, Aleksander S. Popel, Niranjan B. Pandey, Phuoc T. Tran

**Affiliations:** ^1^ Department of Radiation Oncology and Molecular and Radiation Sciences, Sidney Kimmel Comprehensive Cancer Centre, Johns Hopkins School of Medicine, Baltimore, MD, USA; ^2^ Department of Biomedical Engineering, Johns Hopkins University School of Medicine, Baltimore, MD, USA; ^3^ Department of Medical Oncology, Sidney Kimmel Comprehensive Cancer Centre and Department of Urology, The Brady Urological Institute, Johns Hopkins University School of Medicine, Baltimore, MD, USA

**Keywords:** hepatocellular carcinoma, angiogenesis, c-Met, HGF, IGF1R

## Abstract

Hepatocellular carcinoma (HCC) is a major cause of cancer-related death worldwide. Due to inadequate screening methods and the common coexistence of limited functional liver reserves, curative treatment options are limited. Liver transplantation is the only curative treatment modality for early HCC. There are multidisciplinary treatment options like ablative treatments, radiation and systemic therapy available for more advanced patients or those that are inoperable. Treatment resistance and progression is inevitable for these HCC patients. Newer therapeutics need to be explored for better management of HCC. HCC is a hypervascular tumor and many pro-angiogenic proteins are found significantly overexpressed in HCC. Here we explored the therapeutic potential of the anti-angiogenic, anti-lymphangiogenic, and directly anti-tumorigenic biomimetic collagen IV-derived peptide developed by our group. Human HCC cell lines HuH7, Hep3b and HepG2 showed significant disruption of cell adhesion and migration upon treatment with the peptide. Consistent with previously described multimodal inhibitory properties, the peptide was found to inhibit both c-Met and IGF1R signaling in HepG2 cells and blocked HepG2 conditioned media stimulation of microvascular endothelial cell (MEC) tube formation. Furthermore, the peptide treatment of mouse HepG2 tumor xenografts significantly inhibited growth relative to untreated controls. The peptide was also found to improve the survival of autochthonous Myc-induced HCC in a transgenic mouse model. Mechanistically, we found that the peptide treatment reduced microvascular density in the autochthonous liver tumors with increased apoptosis. This study shows the promising therapeutic potential of our biomimetic peptide in the treatment of HCC.

## INTRODUCTION

Liver cancer is one of the top causes of cancer related mortality in the world and the incidence has been increasing in the US for decades [1]. Surgical resection and/or liver transplantation are the curative options for early diagnosed hepatocellular cancers [2]. However, the prognosis for advanced stages of the disease is dismal owing to a lack of chemotherapeutic options. A series of anti-angiogenic drugs have been undergoing clinical trials for some time but they have not had much success because of drug related toxicity and partly because many cases of HCC are refractory to different systemic therapies [3]. Sorafenib, a small molecule inhibitor of multiple kinases, including Raf-1, B-Raf, vascular endothelial growth factor receptors 1-3 (VEGFR1-3), platelet-derived growth factor receptor β (PDGFRβ) and Bcr-Abl and of the non-kinase STAT3, is the only FDA approved treatment option for advanced HCC [4–7]. Sorafenib was found to slow HCC proliferation within the S/G2/M phases of the cell cycle and sensitize the cells to apoptotic stimuli [8]. In addition, inhibition of VEGFR2 and PDGFRβ disrupts angiogenesis, thereby limiting nutrient and oxygen availability to the tumor and minimizing metastatic dissemination. However, the overall survival benefit of sorafenib was a modest improvement of only 2 to 3 months while side effects related to drug toxicity were clinically significant [9]. Therefore, the development of new, more effective systemic therapies for HCC remains an unmet medical need.

Compared to normal liver cells, HCC tumor cells are often associated with the dysregulation of numerous cellular signaling pathways. Expression changes or mutations of growth factor signaling components, specifically c-Met and insulin-like growth factor 1 receptor (IGF1R) are well documented in the pathogenesis of HCC [10]. Overexpression of c-Met and IGF1R is observed in 20-48% or 33% of HCC tumors, respectively, as are increases in the expression of the IGF1R ligand IGF-II [11]. Downstream effectors of insulin receptor substrates 1 (IRS-1) [12] and 2 (IRS-2) [13] have likewise been reported to be overexpressed in HCC. The increased activation of these receptor pathways is thought to negatively impact disease outcome through their stimulation of proliferation, survival, and metastasis [14–16]. Given their importance in HCC, several preclinical and clinical trials targeting these signaling pathways have been completed or are currently underway. Notably, a phase II clinical trial of the c-Met inhibitor tivantinib was found to significantly improve overall survival in patients with c-Met positive HCC and is currently undergoing a phase III trial alone and a phase I trial in combination with sorafenib [17]. Several phase II trials for anti-IGF1R monotherapy are also ongoing, but completed trials have yet to report any significant benefit [18]. Alternatively, IGF1R inhibition may increase the sensitivity of HCC tumors to other compounds and several additional phase II trials are in their early stages investigating the effects of IGF1R-targeted treatments in combination with sorafenib, regorafenib or erlotinib. Currently, regorafenib, a tyrosine kinase inhibitor approved as a second line therapy for gastrointestinal stromal tumors and metastatic colorectal cancer, is undergoing phase III clinical trials for advanced HCC tumors that progress under sorafenib [19] However, median survival is only increased by 3 months, justifying the need for continued research into alternative therapies.

In addition to changes in growth factor receptor pathways many HCC tumors exhibit enhanced expression of *c-myc* [20]. This proto-oncogene encodes a transcriptional regulator that influences the transcriptional status of as much as 15-20% of the human genome. Notably, c-Myc activity is closely associated with the cell cycle, metabolism, and apoptosis and c-Myc overexpression in cancer is correlated with increased proliferation and glycolytic metabolism [21, 22]. Likewise, increased c-Myc alters the expression of secreted factors in HCC cells which can lead to changes in the tumor microenvironment that facilitate tumor progression, including the induction of angiogenesis and the activation of pro-fibrotic and HGF-secreting hepatic stellate cells [23, 24].

Our group previously described several classes of novel anti-angiogenic peptides that have anti-cancer properties [25–28] The treatment of blood and lymphatic endothelial cells with these peptides was found to disrupt their adhesion and migration as well as inhibit the phosphorylation of important angiogenic and lymphangiogenic growth factor receptors, such as VEGFR2, VEGFR3, c-Met, IGF1R, and PDGFRβ as in [29]. One such biomimetic peptide derived from collagen type IV was demonstrated to inhibit the growth and metastasis of triple negative breast cancer xenografts in mice; the peptide is described in detail in [29] and is referred in the present study as AXT050. Mechanistically, the peptide was found to bind to α_5_β_1_ and α_v_β_3_ integrins, which are important for the dynamic formation of focal adhesion complexes involved in adhesion and migration and signaling complexes with growth factor receptors. Here we describe the therapeutic potential of AXT050 against HCC using *in vitro* and *in vivo* pre-clinical models. Employing various human HCC cell lines we demonstrate that the peptide is able to inhibit the adhesion, migration, and signaling of the cancer cells directly as well as disrupt their ability to influence the activities of nearby endothelial cells. These investigations were extended into mouse xenografts using HepG2 cell line where the findings demonstrated significant tumor growth delay on treatment with AXT050. Furthermore, we observed that AXT050 treatment improved the survival of autochthonous c-Myc oncogene driven HCC.

## RESULTS

### The AXT050 peptide disrupts cellular adhesion and migration in liver cancer cell lines

**Figure 1:**
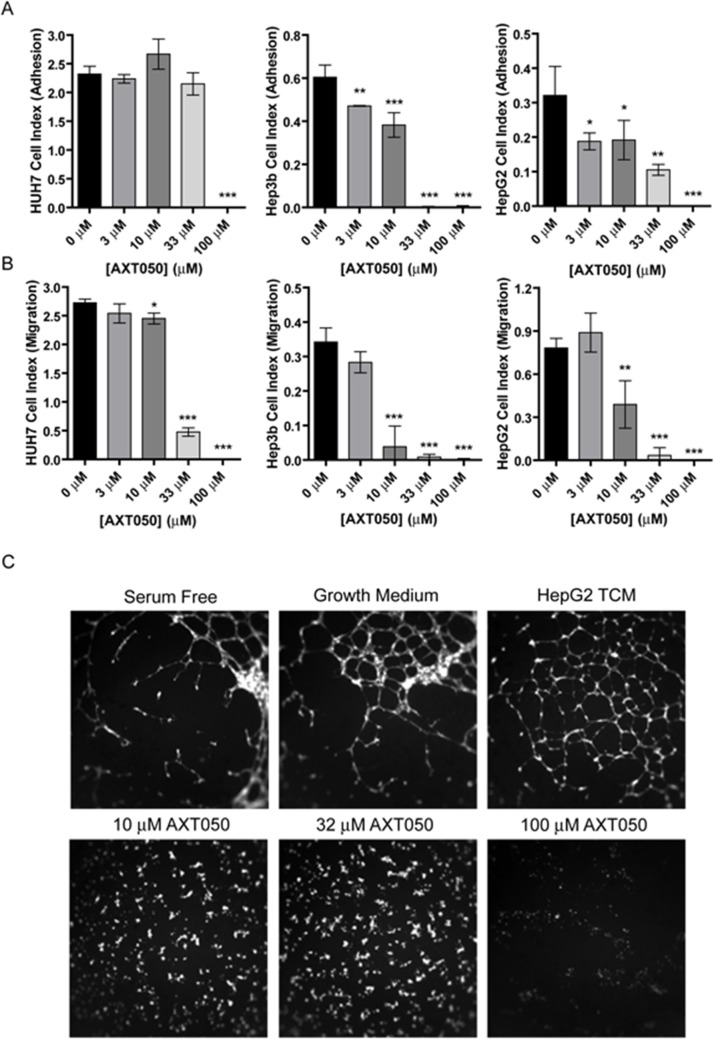
*In vitro* effects of AXT050 on liver cancer cells **(A)** AXT050 significantly reduced the adhesion of HuH7, Hep3b and HepG2 HCC cell lines. Data presented as mean ± SEM; HuH7 (p = <0.0001), Hep3b (p = <0.0001), and HepG2 (p = 0.0001) by 1-way ANOVA. ^*^, ^**^, or ^***^ designate p values ≤ 0.05, 0.01, or 0.001 respectively by Tukey test for differences compared to the 0 μM AXT050 samples. **(B)** AXT050 significantly inhibited the migration of HuH7, Hep3b and HepG2 HCC cell lines. Data presented as mean ± SEM; HuH7 (p = <0.0001), Hep3b (p = <0.0001), and HepG2 (p = <0.0001) by 1-way ANOVA. ^*^, ^**^, or ^***^ designate p values ≤ 0.05, 0.01, or 0.001 respectively by Tukey test for differences compared to the 0 μM AXT050 samples. **(C)** AXT050 treatment (10 μM, 32 μM, and 100 μM) completely inhibited tube formation by HMEC incubated in HepG2 tumor conditioned media.

Previously, AXT050 was shown to disrupt the adhesion and migration of microvascular endothelial cells and lymphatic endothelial cells *in vitro*, as well as viability of different types of breast cancer cells [29]. To better understand how AXT050 influences liver cancer cells directly, we treated three human hepatocellular carcinoma cell lines, HuH7, Hep3b, and HepG2, with various concentrations of peptide and monitored the adhesion and migration of these cells using the ACEA real time cell analysis (RTCA) system. Similar to endothelial cells, increasing concentrations of AXT050 (0 μM, 3 μM, 10 μM, 33 μM and 100 μM) were found to reduce the adhesive properties of all three cell lines (Figure 1A). These reductions were significant at concentrations as low as 3 μM for the Hep3b and HepG2 but only observed at 100 μM for HuH7. In comparison, migration in all three cell lines was significantly inhibited starting at 10 μM AXT050 (Figure 1B). The effect of the peptide on proliferation was also assessed for each cell line (Supplementary Figure 1). In contrast with adhesion and migration, proliferation showed no significant differences at all tested concentrations.

### The AXT050 peptide blocks angiogenesis induced by HepG2

Our earlier work has demonstrated that AXT050 treatment potently inhibits vascular growth in cellular and mouse models of angiogenesis [26, 29]. However, these assays have been performed using specific growth factors or cell lines derived from non-hepatic tissue. In comparison HCC cells are known to secrete a variety of pro-angiogenic growth factors and cytokines [30], which may demonstrate greater angiogenic potential than isolated factors. To investigate the ability of the peptide to inhibit HCC-mediated angiogenesis, tumor conditioned media (TCM) isolated from HepG2 cultures were used to stimulate microvascular endothelial cell (MEC) tube formation (Figure 1C). The formation of these tubular structures is thought to be indicative of pro-angiogenic conditions. We first investigated the relative amounts of angiogenesis-related factors within the TCM and isolated MECs using antibody profiler arrays (Supplementary Figure 2). The arrays revealed the presence in the TCM of several prominent pro-angiogenic factors, including angiogenin, IL8, IGF1 binding protein, PAI-1, PlGF, urokinase-type plasminogen activator (uPA), and VEGF (Supplementary Figure 2A). Additionally, several factors, including Angiopoietin-2 (Ang2) and CCL2, were secreted by the MECs themselves (Supplementary Figure 2B). Treatment of MECs with this TCM induced the formation of tube-like structures over the entire surface of the well compared to the partial coverage observed with cells cultured in serum free media, demonstrating a clear induction of angiogenesis by the HepG2 secretome. This HepG2 TCM-induced MEC tube formation was completely inhibited at all concentrations of peptide tested.

### The AXT050 peptide inhibits HGF- and IGF1-activated signaling in HepG2 cells

**Figure 2:**
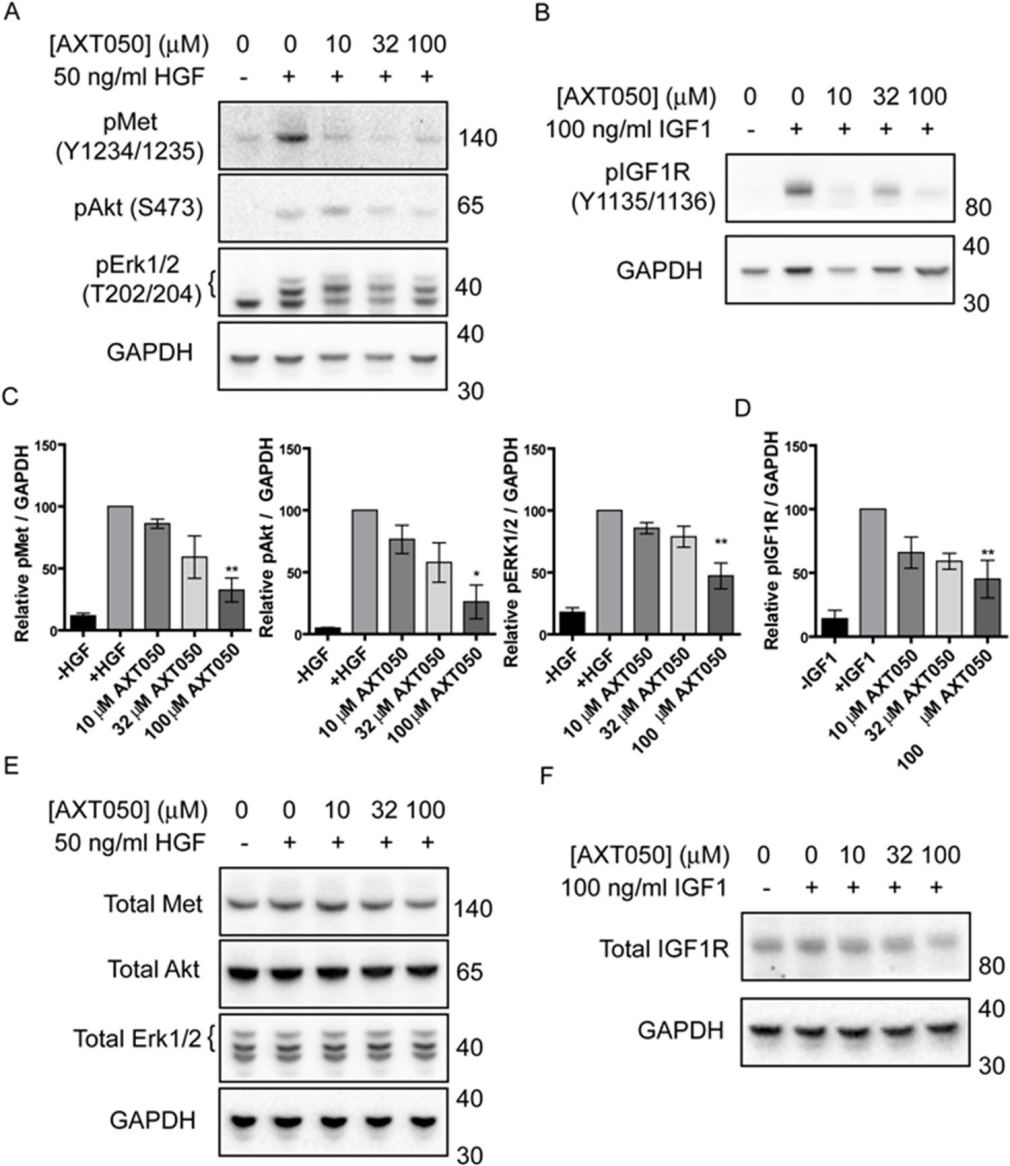
AXT050 inhibits IGF1R and c-Met signaling in HCC cells **(A)** Western blotting demonstrating dysregulation of HGF mediated pMet, pAkt and pErk signaling pathways following treatment of HepG2 cells with AXT050. The presented figures were cropped from images of the original membranes that were cut at approximately 70-75 kDa. The top portion was blotted for pMet and the bottom portion blotted sequentially for GAPDH, pAkt, and pErk 1/2 in that order. Bands for GAPDH are still visible in the pErk 1/2 image (lowest bands) because of this sequential blotting technique. **(B)** Western blotting showing dysregulation of IGF1 mediated IGF1R signaling following treatment of HepG2 cells with AXT050. The presented figures were cropped from images of the original membranes that were cut at approximately 70-75 kDa. The top portion was blotted for pIGF1R and the bottom portion blotted for GAPDH. **(C, D)** Semi-quantitative assessment of band intensity of signals of activated pMet, pAkt, pErk and pIGF1R normalized to GAPDH showing significant downregulation of Met and IGF1R signaling with increasing concentrations of AXT050 (10 μM, 32 μM and 100 μM). Data presented as mean ± SEM (N=3); pMet (p = 0.0009), pAkt (p = 0.0029), pERK 1/2 (p = 0.0002), and pIGF1R (p = 0.0002) by 1-way ANOVA. ^*^ and ^**^ designate significant (< 0.05) and highly significant (<0.01) differences respectively by Tukey test compared to the growth factor, 0 μM AXT050 treated samples. **(E, F)** Representative western blots showing total protein levels of Met, AKT, ERK 1/2 (E) and IGF1R (F) in HepG2 lysates following treatment with AXT050. GAPDH is provided as a loading control. Blots were otherwise prepared as described for phosphorylated equivalents (A and B).

Treatment with AXT050 was found to inhibit the signaling of multiple growth factor receptors in endothelial cell lines [26, 29]. In order to assess if AXT050 influenced the signaling of similar pathways in hepatocellular cancer cell lines we investigated the effect of the peptide on the phosphorylation of c-Met and IGF1R receptors as well as the common downstream mediators of survival, Akt, and proliferation, Erk 1/2, in HepG2 cells following exposure to HGF or IGF1 (Figure 2A–2D). Treatment of HepG2 cells with increasing concentrations of AXT050 decreased HGF-induced phosphorylation of c-Met (Y1234/Y1235), Akt (S473), and Erk 1/2 (T202/Y204) and IGF1-mediated IGF1R phosphorylation (Y1135/Y1136) after treatment relative to samples containing growth factor alone. Trends were dose-dependent in all samples with significance observed at 100 μM. The treatment did not affect the total levels of any of the proteins tested (Figure 2E, 2F).

### Treatment with the AXT050 peptide inhibits tumor growth *in vivo*

**Figure 3:**
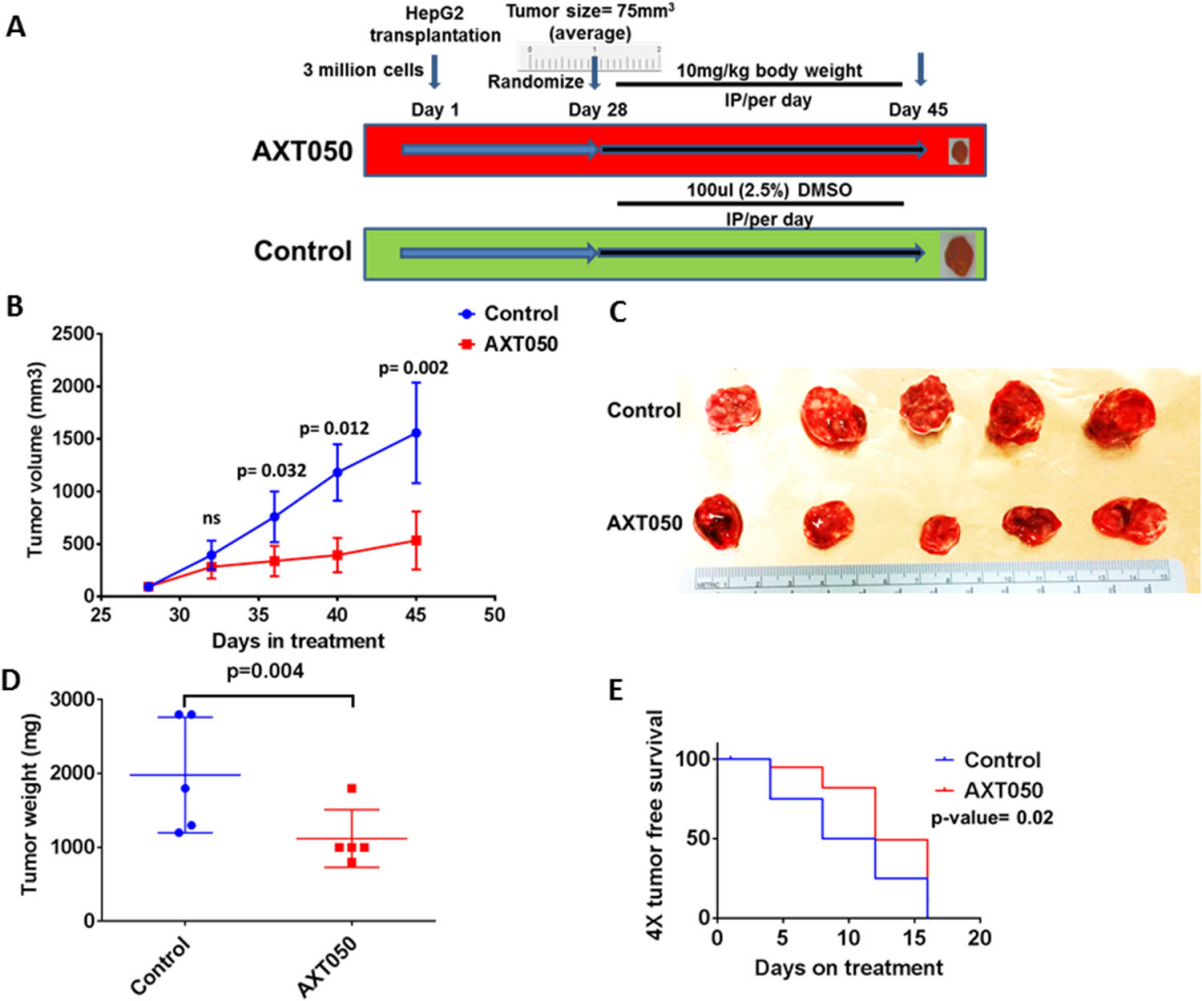
AXT050 treatment *in vivo* delayed tumor growth of subcutaneous HepG2 xenografts **(A)** Treatment schema: Tumors were allowed to grow to a volume of 75 mm^3^, 5 mice were randomly assigned to each of the two groups: (1) no treatment, vehicle control; (2) AXT050 treatment. Mice in the treated arm were given a single dose of 10 mg/kg AXT050 by body weight, intraperitoneally daily. **(B)** Significant tumor growth delay following treatment with AXT050 (at day 48 Mean ± SEM of Control= 797.9 ± 262.9, (n=5) ; Mean ± SEM of treatment= 329.9 ± 72.00, (n=5) p= 0.002). **(C)** Gross tumor volume of control and treatment two weeks following treatment showing reduced tumor size in treatment group. **(D)** Tumor weight is significantly reduced on treatment with AXT050 (Mean ± SEM of Control= 1980 ± 349.9 (n=5); Mean ± SEM of treatment = 1120 ± 174.4, (n=5) p= 0.004). **(E)** AXT050 inhibits tumor growth *in vivo* over four weeks of treatment as graphed by 4x tumor doubling. Kaplan-Meier survival analysis where the event was considered time to tumor quadrupling. AXT050 resulted in statistically significant mean tumor growth delay: AXT050 = 12 days > Vehicle Control = 8 days (p= 0.02).

To assess the effect of the drug on pre-clinical mouse xenografts, HepG2 cell line grafted subcutaneous tumors were grown in athymic nude mice and randomized to the treatment schema shown in Figure 3A. There was tumor growth delay following AXT050 treatment, with a tumor volume growth inhibition of approximately 70% observed when comparing tumors from AXT050 treated animals to tumors from controls on the final day of treatment (see Figure 3B and methods). The mean tumor volume difference between AXT050 treatment and control was observed to be statistically significant during the treatment period of the experiment between day 36 (p=0.032) and day 45 (p=0.002), the final day of measurements and harvest of the tumors (Figure 3B). There was also a significant difference in mean tumor weight between AXT050 treatment and control at the 17^th^ day of treatment, day 45 of the experiment (p= 0.004) (Figure 3C, 3D). The tumor growth inhibition rate (IR) calculated from the gross wet weight of tumors after resection also shows 53% of tumor growth inhibition (Figure 3D). The 4X tumor free survival analysis in AXT050 treated and untreated HepG2 xenograft tumors as measured during treatment also suggested tumor growth delay (AXT050= 12 days > Vehicle control= 8 days; p= 0.005) (Figure 3E).

### The AXT050 peptide treatment improves survival of mice with Myc-induced autochthonous liver tumors

**Figure 4:**
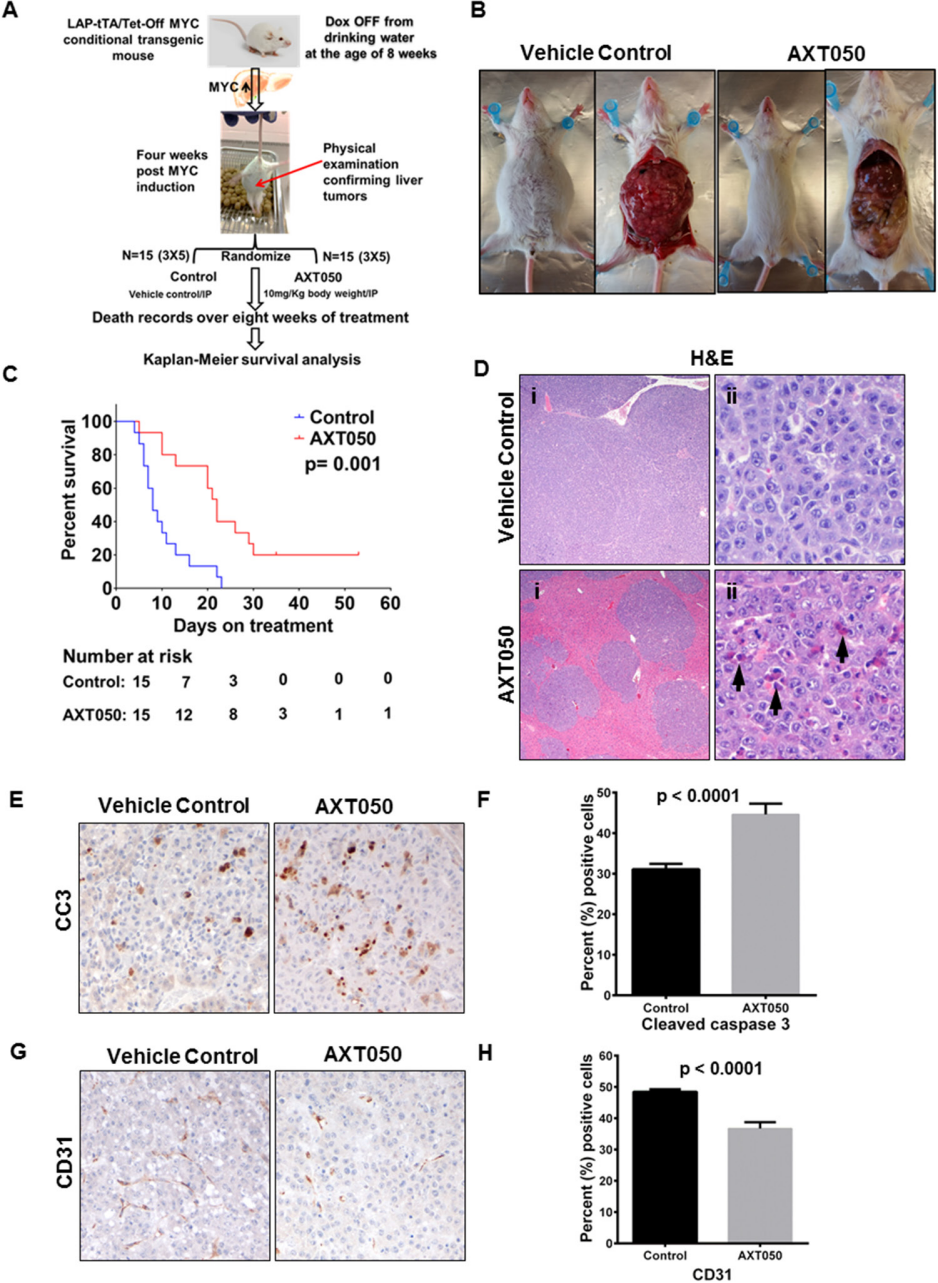
AXT050 improved survival of mice with Myc-induced autochthonous liver tumors, reduced HCC tumor microvascular density, and increased apoptosis **(A)** Treatment schema. Myc oncogene was induced in the mice by removing doxycycline from the drinking water at eight weeks of age. After four weeks physical examinations of the mouse abdomen were conducted for the presence of liver tumors. Upon confirmation of increased abdomen size, presence of ascetic fluid as depicted, the mice were randomized to treatment and control arms. Mice in the treated arm were given a single dose of 10mg/kg AXT050 by body weight intraperitoneally daily. Deaths were recorded for survival analysis. **(B)** Gross pathology showing reduced tumor burden in AXT050-treated mice. **(C)** AXT050 significantly increases the survival of mice with Myc-induced autochthonous liver tumors. Kaplan-Meier survival analysis showing a significant difference in survival of AXT050 treated animals (N=15) vs control (N=15) group of mice. Median survival AXT050 = 22 days > Vehicle Control = 8 days (p= 0.001). **(D)** Extensive replacement of normal hepatocytes by neoplastic cells in vehicle control (i). Treated groups show less extensive tumor infiltration and numerous apoptotic cells (arrowheads, ii). **(E)** Increased apoptosis in treated groups indicated by cleaved caspase 3 immunohistochemistry. **(F)** Histogram showing mean percentage of cleaved caspase 3 positive cells were significantly higher than the control tumors (Mean ± SEM of control= 31.10 ± 0.2982, (n=20) vs Mean ± SEM of AXT050= 44.60 ± 0.5956, (n=20), p< 0.0001). **(G)** Decreased vascular density in treated groups indicated by CD31 immunohistochemistry. **(H)** Histogram showing significantly reduced mean percentage of CD31 stains in the AXT050 treated tumors compared to control tumors (Mean ± SEM of control = 49.20 ± 0.4115, (n=20) vs Mean ± SEM of AXT050 = 37.50 ± 0.8946, (n=20), p< 0.0001).

To further study the *in vivo* therapeutic potential of AXT050, we used a Myc-induced autochthonous transgenic HCC mouse model [31] and tested efficacy of the drug on overall survival. Treatment schema was as shown in Figure 4A. On gross pathology, we observed tumor regression in the AXT050 treated group after treatment for two weeks (Figure 4B). Kaplan-Meier survival analysis on 15 treatment mice and 15 control mice treated over a period of eight weeks showed significant increase in the survival of AXT050 treated mice (20% survival with AXT050 vs. 0% survival with control). Wilcoxon log-rank test on the median survival of treatment (N=15) and control (N=15) showed statistically significant increase in the median survival of the treated animals (median survival=8 days in control vs median survival = 22 days in treatment; p= 0.001 (Figure 4C). Hematoxylin and eosin (H&E) staining of different organs including kidney, spleen, lung, pancreas and stomach showed normal morphology in both the treatment and vehicle control groups of mice (Supplementary Figure 3). It is important to note that c-Myc is a driver oncogene for hepatocellular cancer and approximately 25% (89/366) of HCC tumors have MYC amplification, alteration and gain of function mutations. The MYC gene expression in HCC is associated with its gain of function genetic alterations as retrieved from the TCGA-cBioportal database (Supplementary Figure 4) [32, 33]. The pre-clinical findings showing significant improvement in the survival of the MYC induced liver cancer mice indicates the therapeutic potential of AXT050 on the subset of MYC amplified HCC patients.

### The AXT050 peptide inhibits tumor growth *in vivo* is associated with increased HCC apoptosis and impaired vascular density

The pathological features of the MYC-induced liver tumors following vehicle control and AXT050 treatment were assessed by routine hematoxylin and eosin (H&E) staining. Examination of the H&E sections showed high infiltration of tumor cells in the control tumors while there was minimal tumor cell infiltration in the treatment group (Figure 4D). It was also observed in the H&E sections that there were numerous apoptotic cells in the AXT050 treated tumors. Immunohistochemistry (IHC) analysis for cleaved caspase 3 was carried out to further corroborate these pathological findings. Overexpression of cleaved caspase 3 (CC3) in the treated tumors was observed compared to vehicle control confirming that the drug reduces tumor burden by inducing sustained apoptotic signaling (Figure 4E). The quantitation of cleaved caspase 3 stains across the control and treatment group shows a statistically significant increase in the percentage of CC3 positive cells in AXT050 treated tumor sections compared to controls (Mean ± SEM of control= 31.10 ± 0.2982, n=20 vs Mean ± SEM of AXT050= 44.60 ± 0.5956, n=20, p< 0.0001) (Figure 4F). Furthermore, immunohistochemistry (IHC) analysis with the microvascular marker (CD31) showed a reduction in the microvascular density with AXT050 treatment compared to vehicle (Figure 4G). The percentage of CD31 positive stains in AXT050 treated tumors were significantly decreased compared to those in the control tumors (Mean ± SEM of control = 49.20 ± 0.4115, (n=20) vs Mean ± SEM of AXT050 = 37.50 ± 0.8946, (n=20), p< 0.0001) (Figure 4H).

## DISCUSSION

HCC is the fifth most common cancer and second leading cause of cancer death worldwide, but little progress has been made in the development of new treatments for advanced stages of the disease [34]. In this work we demonstrated that the biomimetic peptide AXT050 is able to potently inhibit several aspects of HCC tumor progression *in vitro*, including tumor cell adhesion, migration, and intracellular-signaling. The peptide could also inhibit HCC stimulation of angiogenesis as shown by microvascular endothelial cells exposed to HCC conditioned media. We then extended these efforts *in vivo* using both xenograft and autochthonous transgenic mouse models of HCC and observed significant tumor growth delay and enhanced survival following the peptide treatment.

Angiogenesis is a common feature of many solid tumors and plays a critical role in growth and metastatic progression of HCC. Evidence of this vascular dependence has been made apparent in several studies demonstrating the comparable efficacy of anti-VEGF therapy, alone or in combination with TKIs (ie. erlotinib), in the treatment of advanced HCC relative to sorafenib (reviewed in [35]). More recently aflibercept, a soluble decoy construct containing a fusion of VEGFR1 and VEGFR2 ligand binding domains, was shown to inhibit tumor growth in several mouse models of HCC [36]. Aflibercept specifically inhibits the activities of all VEGF isoforms and PlGF, further emphasizing the likely importance of VEGF in HCC.

**Figure 5:**
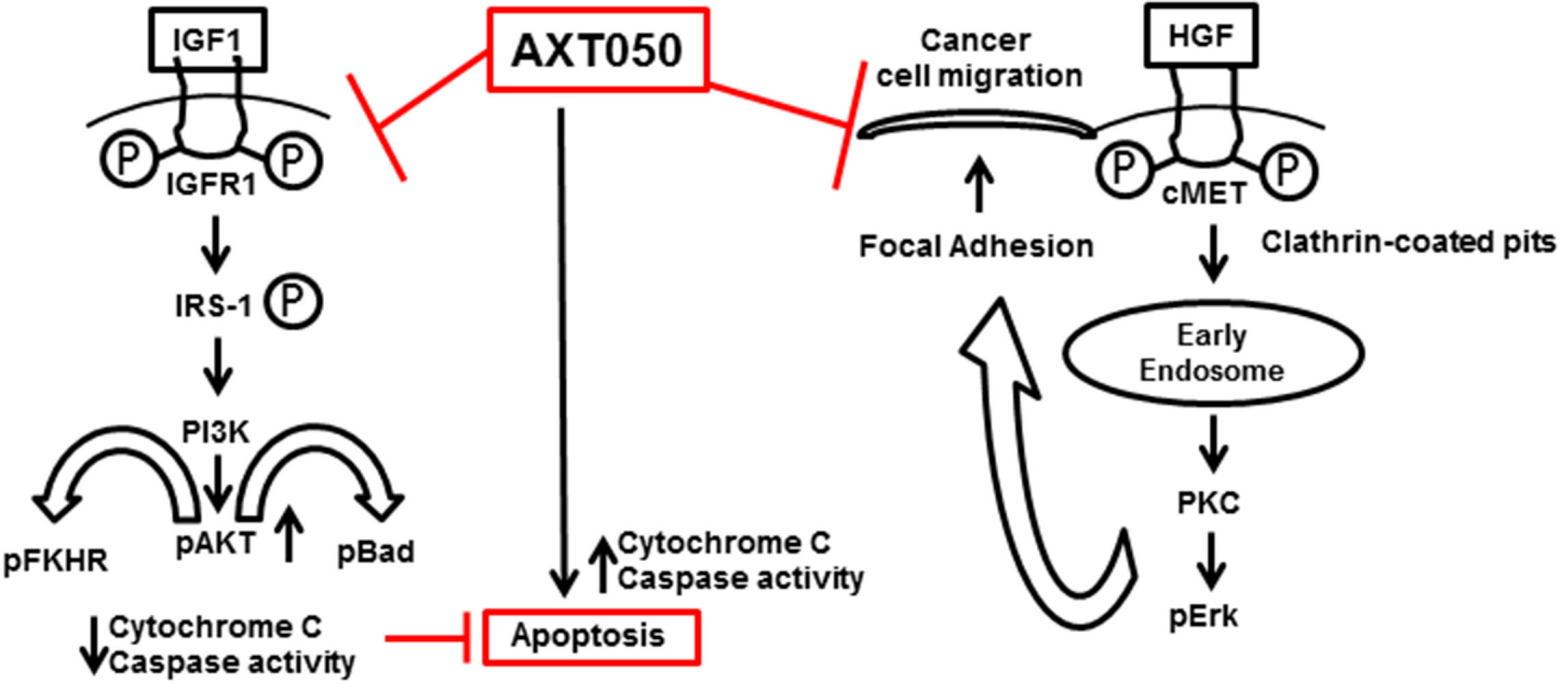
Multimodal anticancer properties and possible mechanism of action of AXT050 in hepatocellular cancer cells *in vitro* (inhibition of cellular migration) and in autochthonous mouse model *in vivo* (inducing apoptosis)

In the present study, AXT050 was found to inhibit the angiogenic stimulation of microvascular endothelial cells by HepG2 secretions *in vitro* and vascularization in autochthonous HCC mouse models. Given the long-standing connection between angiogenesis and cancer, we anticipate that this anti-angiogenic activity contributes significantly to the inhibition of tumor progression by the peptide. Of particular relevance to our autochthonous model, increased Myc levels are observed in many cases of HCC and have been shown to increase the expression of VEGF and other angiogenic cytokines [20, 37]. As with bevacizumab, aflibercept, and sorafenib, AXT050 inhibits VEGF-mediated signaling [29]. Moreover, AXT050 shares the multi-target functionality of sorafenib against other growth factor receptors, a property that may allow for more robust or longer lasting responses relative to VEGF-specific therapies. Unlike these other molecules, AXT050 alters growth factor signaling indirectly through binding to integrin receptors, notably α_5_β_1_ and α_v_β_3_ (Supplementary Figure 5), which function as co-receptors needed for critical signaling cross-talk with growth factor receptors such as VEGFR2. Interactions with integrins are known to alter the activities of various receptors, including signaling duration, trafficking, and turnover [38–41]. This indirect mechanism and the effect on multiple signaling pathways may allow AXT050 to continue to function in case of resistance to single-target or direct inhibitors of RTK signaling. The dynamic interactions between integrins and cellular substrates have also been well established in the processes of endothelial cell adhesion and migration that are important for angiogenesis. Based on our observations, we propose a model by which the drug may exercise its antitumor effect through IGF1R and cMet signaling (Figure 5). Further studies are required to fully understand the mechanistic details of AXT050 antitumor functions.

Stability is often a concern when using peptide based therapeutics comprised of natural amino acids. However, our results clearly demonstrate that effective levels of AXT050 can be maintained through daily injections of the peptide. As one explanation, our binding data clearly demonstrate low nanomolar K_D_ values for interactions between the peptide and its target integrins. Therefore, this tight interaction may prolong the half-life of the peptide beyond what would normally be observed in solution. As an alternative explanation, we have also observed peptide self-assembly upon addition to media, which may further protect the peptide from proteolytic degradation while allowing for the slow release of active peptide molecules over the duration of treatment. Nonetheless, a complete pharmacokinetic profile for the peptide remains to be determined.

In comparison to previous work focusing on the effects of AXT050 on endothelial cells, the current experiments are the first to demonstrate the direct inhibition of adhesion, migration, and growth factor signaling pathways in human cancer cell lines. However, these results are not completely surprising for two reasons. First, the peptide was found to inhibit the phosphorylation of PDGFRβ in 3T3 fibroblasts (Supplementary Figure 6), revealing that the effects of the peptide are not limited to endothelial cells. Second, integrin α_5_β_1_ is highly expressed in several HCC cell lines, including HuH7, Hep3b, and HepG2 [42, 43], and integrin α_v_β_3_ is reported at low, but functional, levels in HepG2 cells [44]. Disruption of these integrins would decrease adhesion and migration through the reduction of interactions between cells and extracellular contacts as well as alter the regulation of associated growth factor receptors. As one possibility, the inhibition of adhesion and migration may significantly impact the ability of these cells to metastasize *in vivo*. However, the HCC mouse models used here are not metastatic models. Alternatively, while treatment with AXT050 did not significantly influence the proliferation of HCC cell lines in culture, the reduction in cancer cell migration may have imposed spatial restrictions against dividing cells within the tumor mass *in vivo*, thereby contributing indirectly to the reduced growth rate [45, 46]. The anti-tumor activity of the drug was demonstrated by significant tumor growth delay for the HepG2 subcutaneous xenografts. The autochthonous Myc-induced HCC model also showed significantly extended survival following the peptide treatment. Specifically, AXT050-treatment was associated with sustained apoptosis and decreased vascularization in these models. Furthermore, our data showing the inhibition of TCM-induced endothelial cell tube formation highlights a potential role for AXT050 in the disruption of crosstalk between different cell types within the tumor microenvironment, whether by blocking the effects of secreted cytokines or limiting the infiltration of certain cell types into the tumor (including the endothelial cells themselves). Taken together, the dysregulation of multiple signaling pathways by AXT050, as elucidated by our *in vitro* studies, does indicate that the peptide has potential therapeutic potential for hepatocellular cancer. Nonetheless, exactly how this direct inhibition of cancer cells versus the other effects of the peptide on non-cancer cell autonomous components of the tumor contribute to the total anti-tumor effects we observed remains to be determined.

The dual inhibition of cancer and endothelial cell functions may synergize through the reduction of resistance pathways normally associated with anti-angiogenic therapies. While angiogenesis is important for the exponential growth of cancerous tissue, non-angiogenic processes for tumor vascularization may help bypass the effects of strictly angiogenesis-based therapies. In particular, vessel co-option, or the hijacking of existing vasculature for tumor growth, is observed in models of HCC and has been associated with the diminishing efficacy of extended sorafenib treatment. Tumors from orthotopic mouse models of HCC showed significant preference towards vessel co-option over angiogenic mechanisms following sorafenib administration [47]. Furthermore, in colorectal cancer-derived liver metastases the migration of the invading cancer cells was found to be required for vessel co-option and the dual inhibition of migration and angiogenesis proved more effective than anti-angiogenic therapy alone [48]. Therefore, the combination of anti-angiogenic activity and inhibition of cancer cell migration may increase the efficacy of the peptide over exclusively anti-angiogenic agents and delay the progression of acquired resistance. Finally, the promising anti-tumor activity of AXT050and the favorable side effect profile in our current and previous animal studies [49] suggest that this peptide should be transitioned to early phase clinical studies.

## MATERIALS AND METHODS

### Cell culture and peptide handling

Hepatocellular carcinoma lines, including HuH7 (p53 point mutation), HepG2 (wildtype p53), and Hep3b (p53 and pRb deficient; hepatitis B positive), and 3T3 fibroblasts (American Type Culture Collection (ATCC) Manassas, VA 20108 USA) were maintained in Dubelcco's modified eagle media (DMEM) with 4.5 g/L glucose (Corning, Corning, NY), 10% fetal bovine serum (FBS) (Corning), and 100 U/ml penicillin and streptomycin (Gibco). The liver cancer cell lines were authenticated using short tandem repeat testing and tested negative for mycoplasma using the Johns Hopkins Medicine Genetic Resources Core Facility. Human dermal microvascular endothelial cells (MECs) were purchased form Lonza (Walkersville, MD) and maintained up to passage 8 in EGM-2MV media. AXT050 was produced by solid phase synthesis and purchased from New England Peptide. HPLC and mass spectrometry analysis indicted a purity greater than 90%. The lyophilized peptide was then dissolved in 100% DMSO to a concentration of 40 mM and stored at -20°C until used. For cell-based experiments, aliquots were diluted to 2 mM working stocks in water containing 5% DMSO. Where necessary excess DMSO was added to bring the final DMSO concentration to 0.25% in all samples.

### Cell adhesion and migration assays

Cell adhesion and migration of HuH7, HepG2, and Hep3b cell lines were investigated using the ACEA Biosciences xCELLigence® real time cell analysis (RTCA) system. This system quantifies cellular adhesion by monitoring the increase in electrical impedance resulting from the contact of cells with a layer of gold electrodes within the culture wells. For adhesion assays 5^*^10^5^ cells in DMEM media containing 0 to 100 μM AXT050 and equivalent concentrations of the DMSO vehicle were added to each well of an ACEA E-plate. Electrodes covering the bottom surface of the well then continuously monitor the electrical impedance for at least 3 hours. Migration assays were performed using a CIM plate, with culture wells divided into upper and lower chambers by a cell-permeable membrane. Electrodes covering the lower face of this membrane measure the electrical impedance that follows the migration of cells from the upper to the lower chamber. First, 20 μg/ml fibronectin (Sigma) was added to the upper surface of the membrane and incubated at 37°C for 30 min. Full-serum DMEM medium was then added to the wells of the lower chambers and both halves of the chamber assembled and incubated for an additional 1 h at 37°C. Cells in serum free DMEM media containing 0 to 100 μM AXT050 and equivalent concentrations of the DMSO vehicle were then added to the upper chamber and the electrical impedance monitored continuously for at least 24 h.

### Cell proliferation assays

The proliferation of HCC cell lines were determined using the WST-1 reagent (Roche), which monitors the formation of formazan dye by the cell-mediated cleavage of tetrazolium salt. HuH7, Hep3b, and HepG2 cells were seeded at 1,500 cells per well in clear-bottom, 96 well plates in full-serum, DMEM media and allowed to adhere overnight. The following day the media were carefully removed and replaced with fresh media containing 0-100 μM AXT050. Additional DMSO was added where needed to ensure an equal concentration in all samples (0.25%). Cells were then cultured for an additional 72 h, after which the media were removed and replaced with 1:10 WST-1 in serum and phenol red free medium. Formazan dye formation was allowed to proceed over 4 h and quantified by measuring absorbance at 450 nm using the Victor V fluorescence plate reader (Perkin Elmer). For each plate, all tested concentrations were performed in triplicate and combined with data from three experimental replicates.

### Western blots

HepG2 or 3T3 cells were seeded into 6-well plates or 10 cm diameter dishes and cultured for 24 hours in full serum DMEM media at 37°C and 5% CO_2_. The cells were then serum starved overnight in serum free DMEM media. The next day, cultures were treated with AXT050 or DMSO solution for 90 minutes followed by stimulation with either 50 ng/ml hepatocyte growth factor (HGF) or 100 ng/ml insulin-like growth factor 1 (IGF1) for 15 minutes or 50 ng/ml PDGF-BB for 10 minutes. Cells were then transferred to ice, washed twice with cold dPBS, and lysed in SDS Loading Dye (Cell Signaling Technologies, Danvers MA). Lysates were then sonicated, boiled, and stored at -20°C until needed. Lysates were resolved by SDS-PAGE using 4-12% gradient NuPAGE gel in MOPs buffer (Life Technologies) and transferred to nitrocellulose membranes for Western blotting. Membranes were blocked in 5% bovine serum albumin (BSA) (Sigma-Aldrich, St. Louis, MO) and 5% milk (LabScientific Inc., Highlands, NJ) and incubated overnight with the following primary antibodies (Cell Signaling) in TBST containing 5% BSA and 0.03% sodium azide: phospho-c-Met (Y1234/1235) (Cat#: 3077), c-Met (Cat#: 8198), phospho-IGF1Rβ (Y1135/1136) (Cat#: 3024), IGF1Rβ (Cat#: 3018), phospho-Akt (S473) (Cat#: 4058), Akt (Cat#:9 272), phospho-ERK1/2 (T202/Y204)) (Cat#: 4370), ERK1/2 (Cat#: 4695), phospho-PDGFRβ (Y751) (Cat#: 4549), and GAPDH (Cat#: 2118). Bands were detected by chemiluminescence using HRP-conjugated secondary goat anti-rabbit and sheep anti-mouse antibodies (GE healthcare) diluted in 5% milk in TBST.

### HepG2 tumor conditioned media and antibody arrays

HepG2 tumor conditioned media (TCM) were generated as follows. HepG2 cells (7.5^*^10^6^ cells) in full serum DMEM media were added to a T175 flask and cultured for 72 hours with daily media exchanges. The cells were then washed twice with dPBS containing Ca^2+^ and Mg^2+^ and incubated with 8 ml of serum free DMEM, high glucose media for 24 hours to allow tumor cells to secrete factors. The conditioned media was then collected and stored at -80°C. The contents of the TCM were identified using the Proteome Profiler Antibody Array Kit for Angiogenesis (R&D Systems, Minneapolis, MN) according to the manufacturer's instructions.

### Endothelial tube formation assays

Tube-formation assays were performed using modifications to standard procedures described previously [50]. Matrigel (Corning) was thawed overnight, on ice at 4°C and 50 μl transferred to the desired wells of a 96 well, clear bottom plate. The matrigel was then allowed to solidify at 37°C for at least 30 minutes before use. A flask of MECs cultured to 90-100% confluency in EGM-2MV media were then trypsinized, separated into two tubes, and pelleted by gentle centrifugation. The media was aspirated from each tube and the cells from one tube were resuspended in HepG2 TCM and the other in serum free DMEM as a negative control. Cells were mixed with peptide or DMSO solutions as indicated (DMSO was used for the negative control) and 15,000 cells plated in each of the matrigel coated wells. The plate was then incubated at 37°C in a cell culture incubator for 18 hours after which the cells were fixed in 10% neutral buffered formalin. Images were taken using an Olympus IX81 with a 2x objective and a Hamamatsu Photonics C9100-02 EMCCD camera. As treatment at all concentrations of peptide completely disrupted tube formation, quantification of tube networks was unnecessary.

### Subcutaneous xenografts of HepG2 cells

Athymic nude mice (Harlan, Indianapolis, USA) were maintained under pathogen-free conditions and given food/water according to the Johns Hopkins Animal Care and Use Committee guidelines. Mice were injected subcutaneously in the flank region with 3×10^6^ HepG2 cells in 100 μL of PBS and Matrigel (Invitrogen) mixed 1:1. Once tumors reached a volume of 75 mm^3^, 5 mice were randomly assigned to each of the two groups: (1) no treatment, vehicle control; (2) AXT050 treatment. Mice in the treated arm were given a single dose of 10mg/kg AXT050 by body weight, intraperitoneally daily (Treatment Schema Figure 3A). The tumor volumes were measured (using caliper) three times a week, until the tumors reached the limiting size specified by the Animal Care and Use Committee. The tumor volume was calculated as [Tumor Volume= (Breadth x Breadth) x Length/2]. Relative tumor volume (RTV) was calculated by measuring the mean tumor volume at the final day of the treatment for each condition (day 17 of treatment) and dividing this by the mean tumor volume at day 0 of the treatment. Tumor volume growth inhibition was calculated as: 1 – Mean RTV of Treatment/ Mean RTV of Control. The tumor growth inhibition rate (IR) was calculated using the formula IR (%) = (1 – Mean Tumor Weight of Treatment (800 gm) / Mean Tumor Weight of Control (1700 gm) x 100).

### Autochthonous Myc-induced liver cancer model

We used the LAP-tTA/TetO-Myc (LM) inducible transgenic mouse model previously described [31]. A schematic of the mouse model is provided in Supplementary Figure 7. The Myc oncogene was induced in these mice by removing doxycycline from the drinking water at eight weeks of age. Four weeks after the removal of doxycycline from the drinking water, physical examinations of the mouse abdomen were conducted for the presence of liver tumors. Upon confirmation of increased abdomen size as depicted in Figure 4A, the mice were randomized to treatment and control arms. Another cohort of LM tumor moribund mice were randomized and a short term treatment of two weeks was carried out followed by necropsy of animals to harvest tissue samples for pathological examinations and IHC analysis. All the procedures were carried out according to the Johns Hopkins Animal Care and Use Committee guidelines and with approved protocols.

### Necropsy, histology and immunohistochemistry

Necropsy was carried out on animals from both the subcutaneous and autochthonous models to harvest different organs and compare the histological features of the kidney, spleen, pancreas and stomach in controls versus AXT050 treatment animals. In brief, the different tissues obtained were fixed in 10% buffered formalin (Formalin, Buffered, 10% (Phosphate Buffer/Certified), Fisher Chemical, USA) and submitted to the Johns Hopkins Medicine Oncology Tissue Services for preparation of paraffin blocks and progressive staining with hematoxylin and eosin using standard protocols.

The corresponding paraffin sections were also processed for IHC on the subcutaneous xenograft tumors and following short-term treatment of autochthonous LM HCC tumors. Immunostaining was carried out using an anti-CD31 antibody from Abcam (ab28364, rabbit polyclonal antibody) at a dilution of 1:100. In addition, Rabbit monoclonal antibody for cleaved caspase-3 ((Asp175) (D3E9) CST #9579) from Cell Signalling Technology was used at 1:200 dilution for immunostaining. Detection of the signal was carried out using the Poly-HRP anti-Rabbit IHC Detection Systems (Leica Biosystems, USA) and DAB chromogen system (Vector Labs, USA). The immunostained slides were counterstained with Hematoxylin QS (H-3404, Vector Labs, USA). At least 5 tumor fields from immunohistochemistry stains were randomly selected from each of the control (n=4) and AXT050 treated (n=4) animals. Thus twenty (20) random tumor fields from four (4) different mice in each group were scanned in each group to observe any statistically significant difference in the staining. The DAB chromogenic stains and nuclear stains (hematoxylin) were counted using color deconvolution IHC tool box of the Image J, the open source image analysis tool. The percentages of positive cells (DAB chromogens) were calculated in treatment and control sections. The student t-test was carried out using the GraphPad Prism version 5.0 software to compare the mean (percentage of positive cells) in two groups for any statistically significant difference.

### Fluorescence anisotropy

FAM-labeled AXT050 (10 nM) in DMSO (final concentration 0.0005%) was incubated on ice with 0 nM -75 nM soluble, recombinant α_5_β_1_ and α_v_β_3_ heterodimeric integrins (R&D Systems) for 30 minutes in 50 mM Tris, pH 7.5; 1 mM CaCl_2_; 1 mM MgCl_2_, and 100 mM NaCl). Changes in fluorescence anisotropy were measured using the Tecan Safire II microplate reader (Tecan) using vertically polarized light at 470 nm and detecting both vertically and horizontally polarized light at 540 nm. The data were normalized by subtracting the average value for anisotropy at 0 nM integrin from each data point and dividing by the graphically determined plateau. Values were plotted using GraphPad Prism version 5.0 software and fit to the following equation (1) for ligand-concentration dependent anisotropy that accounts for receptor depletion:

$$\begin{array}{l} r\\ =r_{f}\\ +\left(r_{b}-r_{f}\right)\left(\frac{\left(K_{D}+\left[L\right]+\left[R\right]\right)-\sqrt{{\left(-\left[L\right]-K_{D}-\left[R\right]\right)}^{2}-4\left[L\right]\left[R\right]}}{2\left[L\right]}\right) \end{array}$$ (1)

where r is the calculated anisotropy, [L] is the concentration of FAM- AXT050, [R] is the concentration of integrin, and r_f_, r_b_, and K_D_ are the best fit values for the anisotropy of free FAM-AXT050, anisotropy of integrin-bound FAM-AXT050, and the equilibrium dissociation constant of the integrin and FAM-AXT050 interaction respectively.

### Statistical analysis

Cell adhesion, migration, proliferation, and Western blot data were analyzed by ANOVA in experimental triplicates. The paired Student's t-test was carried out to compare tumor growth delay and analyze statistical difference between mean tumor volume in tumor growth delay and mean tumor weight at the end of the treatment. The statistical difference in Kaplan Meier survival curve between control and AXT050 treatment animals was assessed by the log-rank (Mantel-Cox) test and the Gehan-Breslow-Wilcoxon test. All the statistical analysis were carried out using the Graphpad Prism v 5.0 package unless mentioned otherwise in the methods.

## SUPPLEMENTARY MATERIALS FIGURES AND TABLES


