# Supplementary material for: Therapeutic potential of an anti-angiogenic multimodal biomimetic peptide in hepatocellular carcinoma
Source: Oncotarget. 2017 Sep 21;8(60):101520–34. doi: 10.18632/oncotarget.21148 (PMC5731893; doi:10.18632/oncotarget.21148)
Supplement: Supplementary file 1 [file oncotarget-08-101520-s001.pdf]

Therapeutic potential of an anti-angiogenic multimodal biomimetic peptide in hepatocellular carcinoma

SUPPLEMENTARY MATERIALS

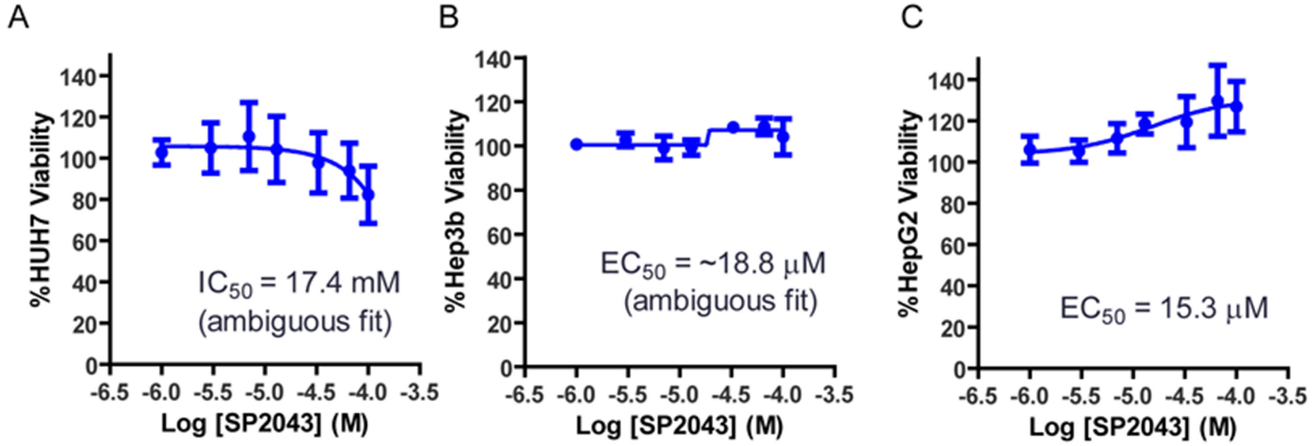

Supplementary Figure 1: Effect of AXT050 on liver cancer cell proliferation. (A-C) AXT050 has no significant effect on cell viability (A) HuH7, (B) Hep3b, and (C) HepG2. Data presented as mean ± SEM; N=3.

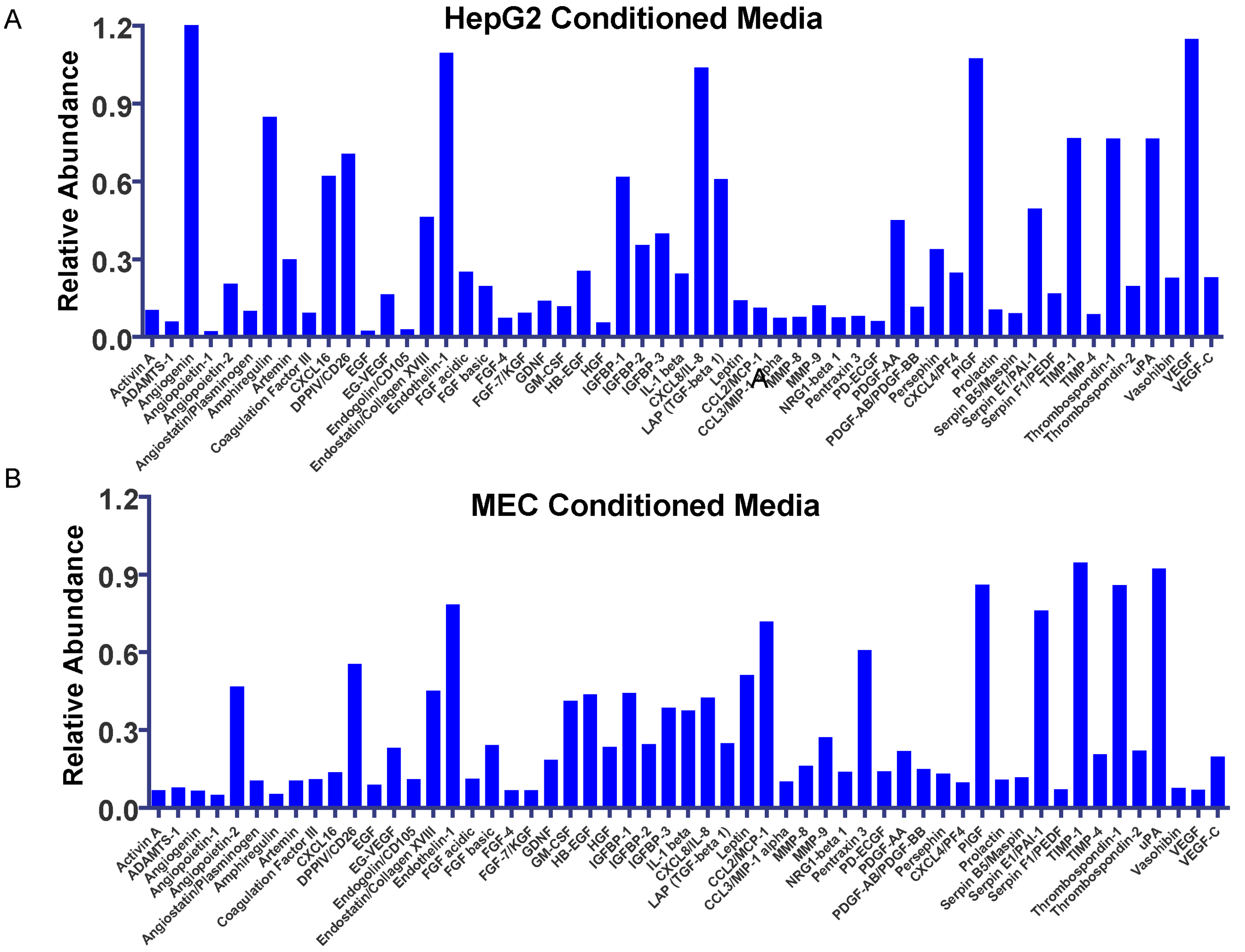

Supplementary Figure 2: The Identification of angiogenic factors in HepG2 and MEC conditioned media. (A, B) Quantification showing the relative abundance of angiogenic factors using antibody arrays exposed to conditioned media from (A) HepG2 or (B) MEC cells.

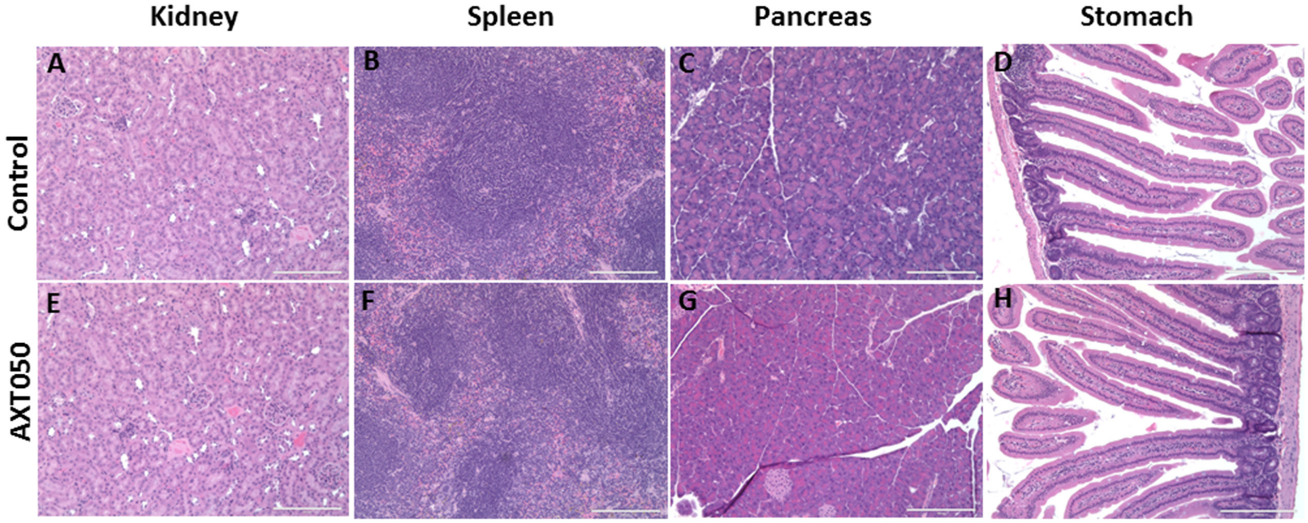

Supplementary Figure 3: Pathological findings on different organs post necropsy showing normal tissue morphology in different organs. Representative images of hematoxylin and eosin sections of various organs on necropsy from control **(A-D)** and AXT050 **(E-H)** treated animals. (A, E) Kidney. (B, F) Spleen. (C, G) Pancreas. (D, H) Stomach.

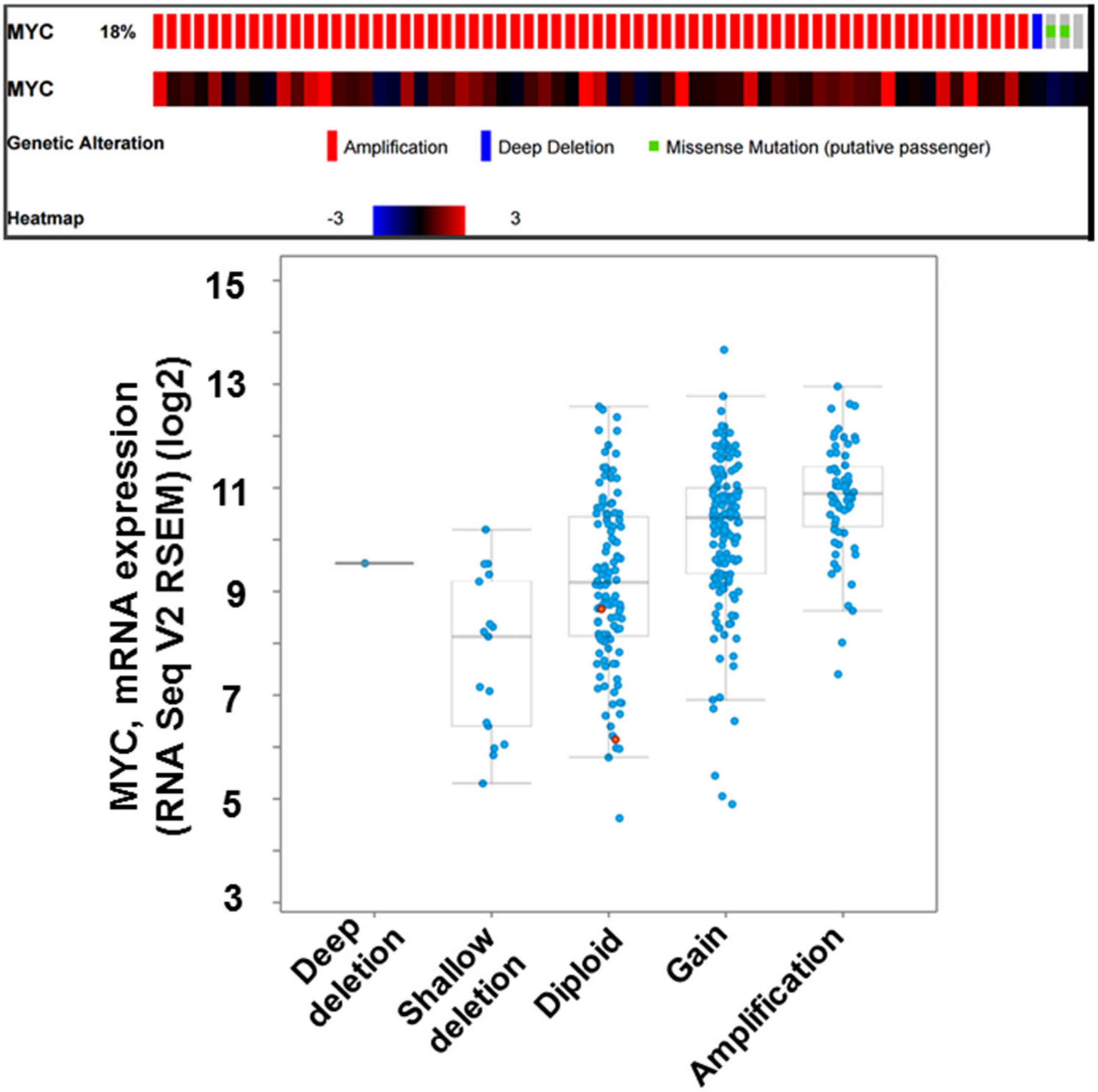

Supplementary Figure 4: MYC gene alterations in HCC. TCGA data analysis using c-Bioportal showing MYC over expression and corresponding gain of function genetic alterations in HCC.

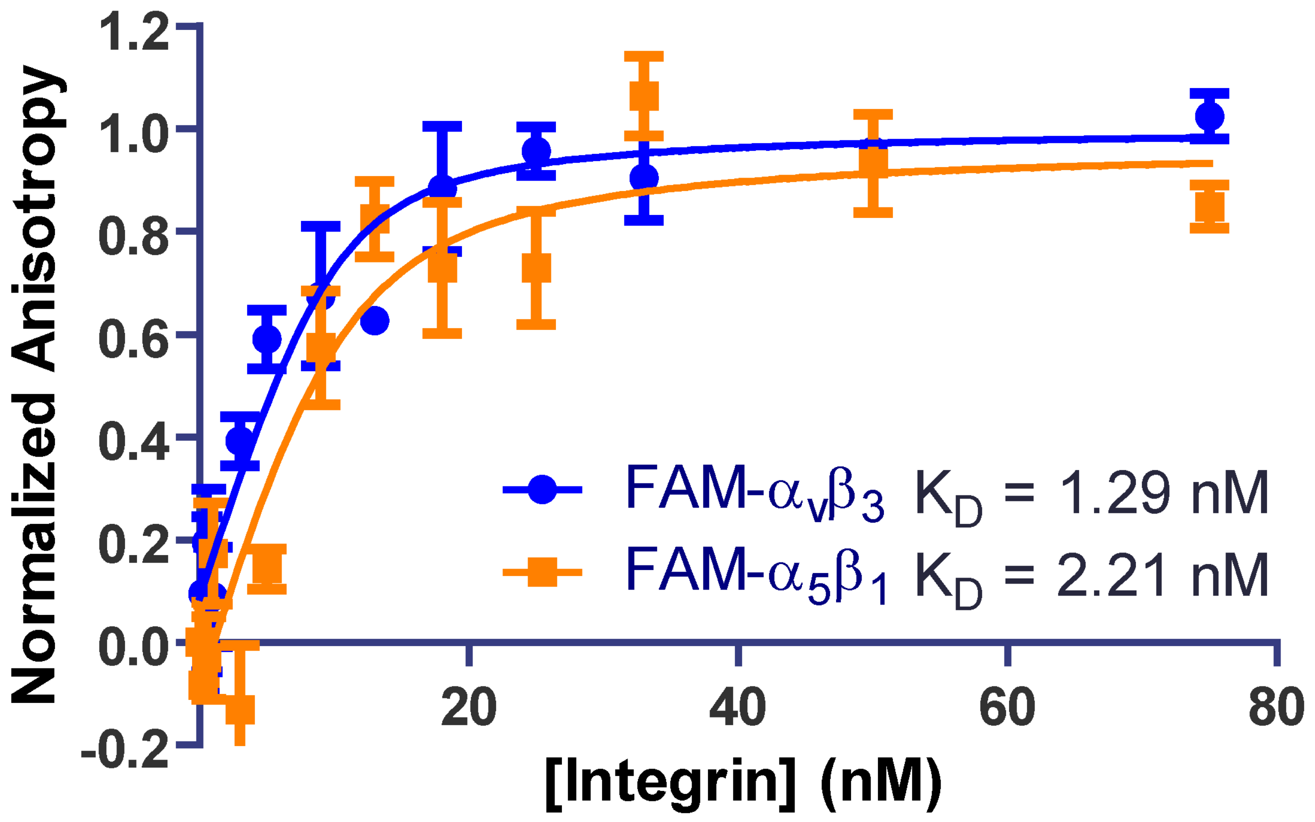

Supplementary Figure 5: AXT050 binds αvβ3 and α5β1 integrins. The normalized change in fluorescence anisotropy for 5-FAM-labeled AXT050 (10 nM) in response to varying concentrations of recombinant αvβ3 (blue) and α5β1 (orange) integrins.

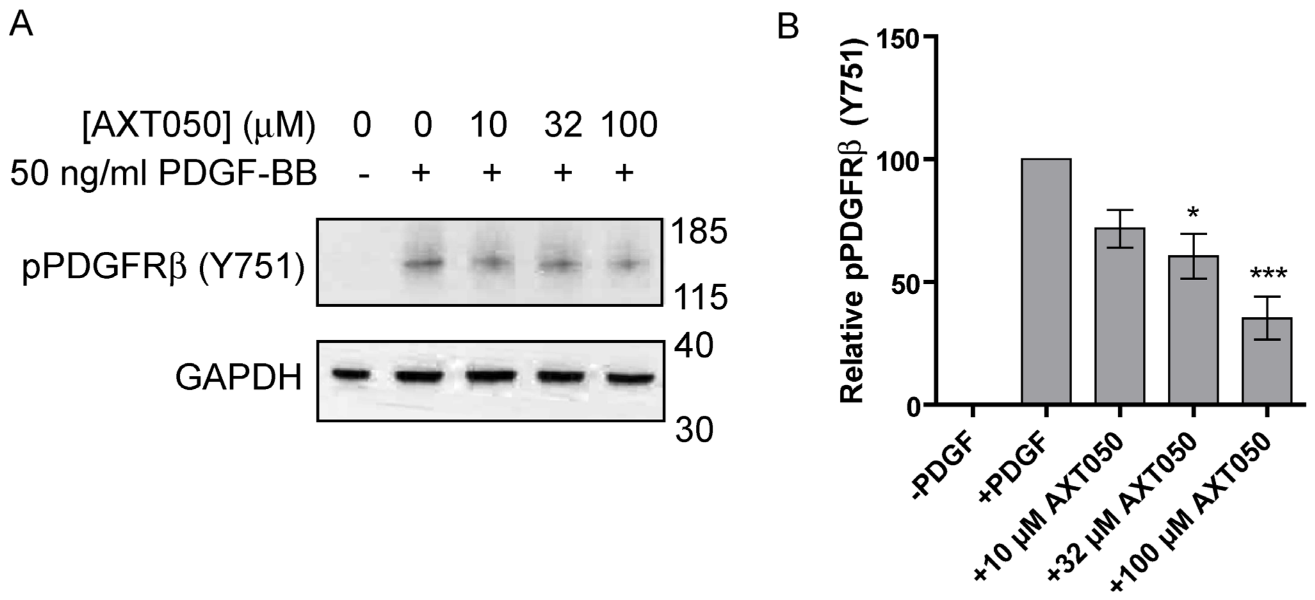

Supplementary Figure 6: AXT050 inhibits the phosphorylation of PDGFRβ. **(A)** Western blotting showing dysregulation of PDGF-BB mediated PDGFRβ signaling following treatment of 3T3 fibroblasts with AXT050. **(B)** Quantification of phospho-PDGFRβ western band intensities normalized to GAPDH bands. Data presented as mean ± SEM (N=3); p = 0.0001 by 1-way ANOVA. * and *** designate significant (< 0.05) and highly significant (<0.001) differences respectively by Tukey test compared to the growth factor, 0 μM AXT050 treated samples.

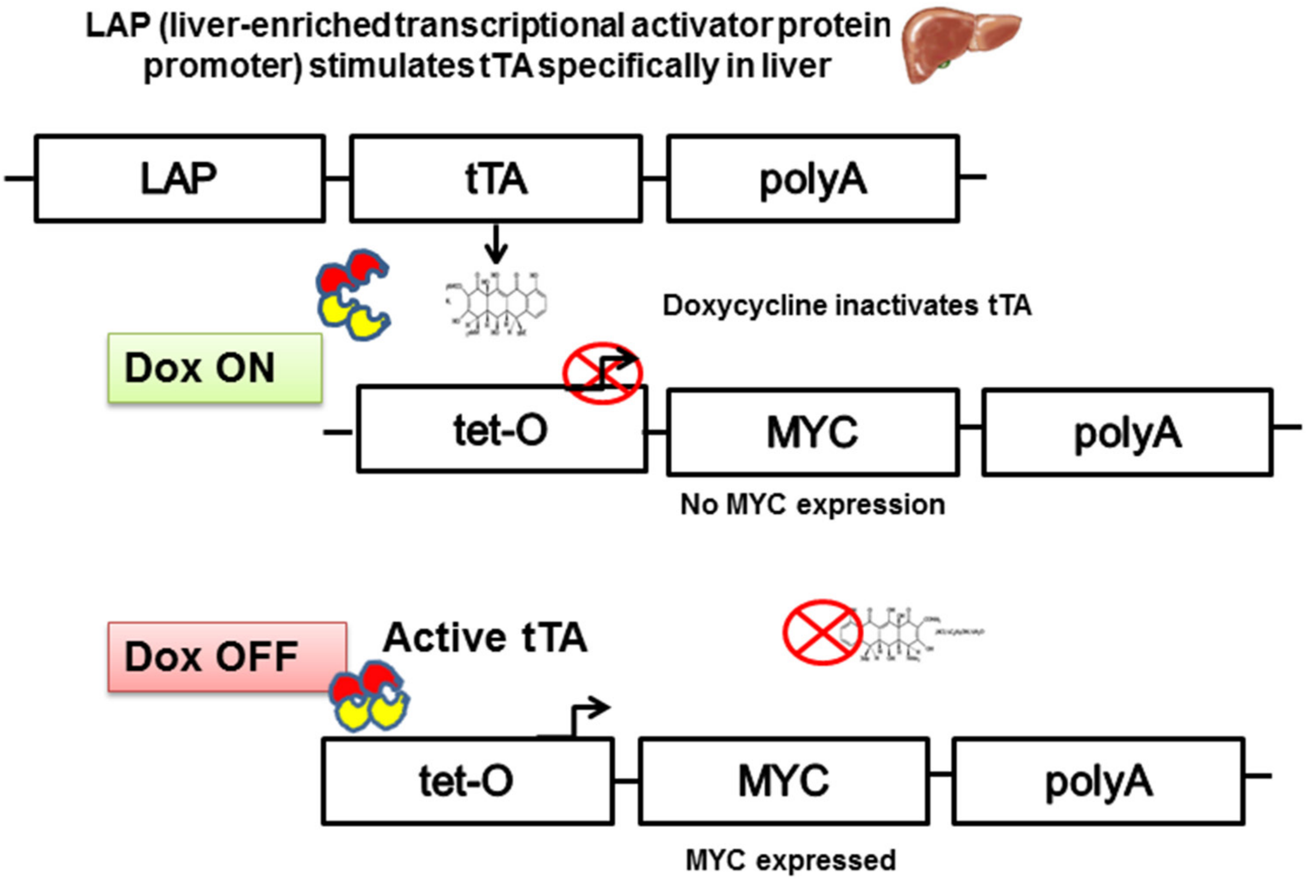

Supplementary Figure 7: Schematic of the autochthonous MYC induced liver cancer mouse model.
